# Report of the Third Hyderabad Chloroform Commission

**Published:** 1893-12

**Authors:** 


### EDITORIAL.

The office of THE JOURNAL is in room 330 Equitable Building.

Address all communications, and make all remittances payable to THE ATLANTA MEDICAL AND SURGICAL JOURNAL, Atlanta, Ga.

Articles for publication should reach this office not later than the 15th instant.

The editors are not responsible for views expressed by contributors.

### REPORT OF THE THIRD HYDERABAD CHLOROFORM COMMISSION.

Within the last few years the medical profession has been much interested in the reports of the commissions that have been appointed by his Highness, the Nizam of Hyderabad, to study the influence of chloroform upon the respiration and circulation. These reports enunciated certain principles which were so much at variance with the established faith that, naturally enough, they attracted universal attention and received liberal comments, both favorable and unfavorable.

It was hard for the profession to believe that "death from chloroform is never due to cardiac failure," and that "ether is as dangerous as chloroform, if given sufficiently to produce true anesthesia." Counter-experiments were made by gentlemen thoroughly competent to observe and interpret the phenomena of anesthesia, and results were obtained and published which were not in harmony with those announced by the commissions.

In March, 1892, Dr. Hobart A. Hare, Professor of Materia Medica in the Jefferson Medical College, of Philadelphia, was requested to institute a third chloroform research. In his work he received the assistance of Dr. E. Q. Thornton. Their report is before us (*Therapeutic Gazette*, October, 1893).

Certain points in regard to the action of chloroform are assumed to be settled: (1) that given in ordinary therapeutic quantity it acts as a powerful and constant depressant to arterial pressure; (2) that a fall of blood pressure always occurs when chloroform anesthesia is produced; and (3) that it exerts a powerful depressant, paralyzant action on the respiratory center. The statement of the previous commissions that "chloroform never causes sudden death from stoppage of the heart," is qualified by adding *unless respiration ceased primarily.* This applies to the healthy heart, but Drs. Hare and Thornton believe that the stoppage of a diseased heart may take place first. Chloroform kills primarily by respiratory failure, and in excessive dose by inhalation depresses the circulation through the centric vaso-motor apparatus, finally depressing the cardiac muscle itself. This circulatory depression may be so profound that recovery is impossible, even with the most thorough artificial respiration. Therefore we cannot afford to ignore entirely the effect of chloroform on the circulation, and we must not consider the patient in danger of circulatory failure only when the respiration ceases, but *as soon as it becomes abnormal.*

The authors regard chloroform as a safe anesthetic in the majority of cases, if given by a person skilled in its use. In administering it, the respiration must be carefully watched. As soon as enough chloroform has been taken to endanger the circulation the respiration will show some signs of abnormality, either in depth or irregularity. The previous Hyderabad Commission stated that gradual cardiac failure never occurred without producing respiratory changes from the first, and Surgeon-Major Lawrie regards the rapidity and depth of the respiratory movements as the entire key to the situation. Safety under chloroform is assured as long as the heart is kept regular and natural (*i. e.*, in a healthy patient), and as long as these conditions are fulfilled it is impossible to produce anything with chloroform but anesthesia; and the Hyderabad Commission has shown that anesthesia alone is entirely free from risk. (Lawrie.)

It will be seen that there is great unanimity of opinion among many competent and skillful observers on the prime question of the cause of death in chloroform poisoning. The evidence of the commissions, with that of Surgeon-Major Lawrie, is too strong to be resisted. That the lethal effect of large dosage is first manifested on the respiration, and later on the circulation, seems therefore to be practically settled. The lesson is obvious.

